# Supplementary material for: Changes in Organic Acids, Phenolic Compounds, and Antioxidant Activities of Lemon Juice Fermented by Issatchenkia terricola
Source: Molecules. 2021 Nov 5;26(21):6712. doi: 10.3390/molecules26216712 (PMC8588450; doi:10.3390/molecules26216712)
Supplement: Supplementary file 1 [file molecules-26-06712-s001.zip › molecules-1443860-supplementary.pdf]

molecules MDPI

Supplimentary table S1 Sensory evaluation standards of fermented and control lemon juice

Items	Description	Score
Clarity/20	The fruit juice is uniform in texture, clear and without precipitation	16-20
	The fruit juice is slightly clear with a small amount of floccule	11-15
	The fruit juice is uneven in texture with a small amount of precipitation	6-10
	The fruit juice is uneven in texture, cloudy and precipitated	1-5
Fruity/30	Typical lemon flavor, pleasant and gentle aroma	21-30
	Exhibits lemons aroma but a little bit lighter	11-20
	No lemons aroma	0-10
Taste/30	Sweet and sour, refreshing on the mouth, good palatability	26-30
	A little bit tart , average flavor, average palatability	21-25
	Distinctly sour, with no bitter taste	16-20
	Distinctly sour, astringent, bitter	0-15
Overall acceptability /20	High acceptance	16-20
	Average acceptance	11-15
	Poor acceptance	1-10
Total score/100		100

Supplimentary table S2 Pearson correlation matrix of physicochemical properties and antioxidant activity in fermented lemon juice

	Total titratable acidity	Total sugar	Reducing sugar	pH	Total Phenolic	Total flavonoid	$ABTS^{+}$ radical scavenging activity	Total reductive ability
Total titratable acidity	1							
Total sugar	0.789*	1						
Reducing sugar	0.890*	0.928*	1					
pH	-0.924*	-0.568	-0.722*	1				
Total Phenolic	0.812*	0.856*	0.787*	-0.711*	1			
Total flavonoid	0.892*	0.864*	0.963*	-0.690*	0.665*	1		
$ABTS^{+}$ radical scavenging activity	0.832*	0.838*	0.820*	-0.590	0.815*	0.840*	1	
Total reductive ability	0.890*	0.630*	0.758*	-0.802*	0.719*	0.750*	0.835*	1

* Significant correlation at 0.05 level (two-tailed).
